# The Association Between Allergic Rhinitis, Eosinophilic Inflammation, and Postoperative Recurrence in Nasal Polyps

**DOI:** 10.1002/ohn.70176

**Published:** 2026-02-24

**Authors:** Wei Zhong, Shaobing Xie, Haijun He, Hua Zhang, Weihong Jiang, Can Liao, Zhihai Xie

**Affiliations:** ^1^ Department of Otolaryngology–Head and Neck Surgery Xiangya Hospital of Central South University Changsha People's Republic of China; ^2^ Hunan Province Key Laboratory of Otolaryngology Critical Diseases Xiangya Hospital of Central South University Changsha People's Republic of China; ^3^ National Clinical Research Center for Geriatric Disorders Xiangya Hospital of Central South University Changsha People's Republic of China; ^4^ Anatomy Laboratory of Division of Nose and Cranial Base Clinical Anatomy Center of Xiangya Hospital, Central South University Changsha People's Republic of China; ^5^ Hunan Provincial Ecological Environment Monitoring Center Changsha People's Republic of China; ^6^ Hunan University Changsha People's Republic of China

**Keywords:** allergic rhinitis, chronic rhinosinusitis with nasal polyps, eosinophilic inflammation, postoperative recurrence

## Abstract

**Objective:**

Although allergic rhinitis (AR) commonly co‐occurs with chronic rhinosinusitis with nasal polyps (CRSwNP), its impact on tissue endotypes and prognosis remains unclear. This study examines the impact of AR on CRSwNP, focusing on its links to tissue eosinophilic inflammation and postoperative recurrence risk.

**Study design:**

Retrospective cohort study.

**Setting:**

Tertiary referral center.

**Methods:**

A retrospective analysis was conducted on CRSwNP patients who underwent functional endoscopic sinus surgery. Based on the presence of comorbid AR, patients were categorized into AR and non‐AR groups. Baseline clinical data, peripheral and tissue eosinophil levels, and prognosis were analyzed. Multivariate Cox regression and Kaplan‐Meier survival analyses were used to assess associations with postoperative recurrence.

**Results:**

A total of 603 patients with CRSwNP were included; 202 had comorbid AR. Compared with non‐AR patients, the AR group had higher rates of prior surgery and asthma and showed increased tissue eosinophil counts and percentages. During follow‐up, patients who recurred had a higher prevalence of AR, elevated peripheral and tissue eosinophil levels, more prior surgery, and higher Lund‐Mackay scores. Multivariable Cox regression and Kaplan‐Meier analysis identified AR as an independent predictor of postoperative recurrence. Moreover, a high tissue eosinophil burden independently associated with recurrence; within the recurrence cohort, AR patients exhibited significantly higher tissue eosinophil counts and percentages than non‐AR patients.

**Conclusion:**

Comorbid AR identifies a CRSwNP subgroup with marked tissue eosinophilia and increased risk of postoperative recurrence. It independently predicts earlier relapse and shorter recurrence‐free survival, likely by amplifying local eosinophilic inflammation.

Chronic rhinosinusitis with nasal polyps (CRSwNP) is characterized by persistent inflammation of the nasal and paranasal sinus mucosa lasting more than 12 weeks, and is clinically manifested by nasal obstruction, rhinorrhea, and hyposmia or anosmia, which substantially impair patients' quality of life.^1^, ^2^ CRSwNP is etiologically complex and highly heterogeneous. By inflammatory phenotype, it can be divided into eosinophilic and noneosinophilic types.^3^ The eosinophilic phenotype is characterized by dense eosinophilic infiltration and type 2 inflammation and is associated with a more protracted course, poorer response to medical therapy, and a higher rate of postoperative recurrence.^4^, ^5^

Recently, growing attention has focused on comorbidities in CRSwNP because they can reshape inflammatory phenotypes and hinder mucosal repair, thereby influencing postsurgical outcomes.^6^ Among these, allergic rhinitis (AR)—an IgE‐mediated type I hypersensitivity disorder—shares close epidemiological and pathophysiological links with CRSwNP.^7^, ^8^, ^9^, ^10^ Multiple studies report that CRSwNP with concomitant AR tends to present with more severe nasal symptoms, more complex mucosal pathology, and a higher risk of postoperative recurrence.^11^, ^12^ However, findings remain inconsistent and mechanisms unclear, likely owing to small sample sizes, short follow‐up, heterogeneous outcome measures, and population or regional differences.

To address these gaps, we conducted a single‐center retrospective cohort of adult CRSwNP patients who underwent functional endoscopic sinus surgery (FESS) over the past five years with long‐term follow‐up. We systematically compared clinical features, eosinophilic indices, and recurrence between AR and non‐AR groups. By clarifying the contribution of AR to recurrence risk, this study provides evidence for refined risk stratification and individualized postoperative management.

## Materials and Methods

### Study Population and Design

This retrospective study included 603 adult patients with CRSwNP who underwent FESS at our institution between January 1, 2017 and January 31, 2022. All patients met the diagnostic criteria outlined in the European Position Paper on Rhinosinusitis and Nasal Polyps 2020 (EPOS 2020).^13^ The diagnosis of CRSwNP was established based on clinical symptoms, nasal endoscopy findings, and computed tomography (CT) of the paranasal sinuses. Patients were excluded if they met any of the following criteria: (1) incomplete clinical data; (2) history of smoking; (3) presence of nasal or paranasal sinus tumors, or chronic infectious diseases other than sinusitis; (4) use of systemic corticosteroids, antibiotics, or other immunomodulatory medications within one month prior to surgery; (5) absence of postoperative follow‐up data or failure to complete the follow‐up protocol.

Baseline characteristics and clinical data were collected prior to surgery, including age, sex, body mass index (BMI), disease duration, comorbidities, peripheral blood eosinophil count and percentage, tissue eosinophil count and percentage, Lund‐Mackay score, Lund‐Kennedy score, and follow‐up duration. This study was approved by the Ethics Committee of Xiangya Hospital, Central South University (2025081112).

### Diagnosis and Grouping of AR

The diagnosis of allergic rhinitis in this study required the presence of both typical clinical symptoms (eg, sneezing, watery rhinorrhea, and nasal itching) and objective evidence from a positive skin prick test result (wheal diameter ≥3 mm compared with negative control) and/or elevated allergen‐specific IgE levels (>0.35 kU/L).^14^ Patients were then classified into two groups according to AR status: CRSwNP with AR and CRSwNP without AR.

### Quantification of Tissue Eosinophilic Inflammation

Tissue specimens were collected intraoperatively during FESS and immediately fixed in 4% paraformaldehyde. After fixation, samples were processed by graded ethanol dehydration, paraffin embedding, and sectioned at 4 µm. Sections were stained with hematoxylin and eosin (H&E) using standard procedures. For each case, five representative high‐power fields (HPFs; ×400) were randomly selected per section, eosinophils were enumerated in each field, and the mean count was calculated. The eosinophil percentage was defined as: eosinophil % = (eosinophil count/total inflammatory cell count) × 100%, reflecting the degree of local eosinophilic infiltration as previously described.^15^

### Follow‐up and Recurrence Assessment

All patients underwent FESS performed by the same senior surgical team adhering to a standardized protocol. The extent of surgery was systematically individualized based on preoperative CT findings and intraoperative exploration, yet within a unified decision‐making framework. Postoperative management followed a uniform regimen, encompassing a short course of systemic antibiotics, nasal irrigation, topical corticosteroid application, and scheduled endoscopic debridement of the surgical cavity.^16^, ^17^, ^18^

All enrolled patients were observed clinically for a minimum of 3 years. A standardized follow‐up protocol was established in this study, which included scheduled outpatient visits, nasal endoscopic evaluations, and telephone consultations, implemented as described in prior studies. Specifically, nasal endoscopic examinations were conducted at 2 weeks, 1 month, 3 months, 6 months, and 12 months postoperatively, followed by assessments every 6 months thereafter to monitor long‐term outcomes.^19^, ^20^ Recurrence of CRSwNP was defined as the reappearance of typical clinical symptoms, together with objective evidence on nasal endoscopy and/or CT imaging, persisting for at least 2 months despite receiving the previously described rescue regimen of antibiotics and oral corticosteroids.^18^, ^21^ The actual follow‐up duration for each patient was calculated from the date of surgery to the date of the terminal event, defined as follows: (1) For patients with recurrence: the date of postoperative recurrence diagnosis. (2) For patients without recurrence: the date of the last documented clinical follow‐up visit, with the requirement that this date be no earlier than 3 years after surgery.^16^

### Statistical Analysis

Categorical variables were presented as counts and compared using the chi‐square test. Continuous variables with normal distribution were expressed as mean ± standard deviation and compared using the Student's *t*‐test or one‐way analysis of variance. Nonnormally distributed variables were presented as medians with interquartile ranges and compared using the Mann‐Whitney *U* test or Kruskal‐Wallis *H* test as appropriate. Multivariable Cox proportional hazards models were applied to adjust for potential risk factors, with different models constructed to assess the association between AR and postoperative recurrence. Potential confounders were included based on clinical relevance and prior literature. Multicollinearity among covariates was assessed using variance inflation factors (VIF), with all values <5 for these covariates. Regression assumptions were verified, including linearity of continuous variables and independence of observations. Kaplan‐Meier survival analysis was used to evaluate the relationship between AR and the risk of CRSwNP recurrence. A two‐sided *P* < .05 was considered statistically significant. All statistical analyses were conducted using SPSS software version 26.0 (IBM).

## Results

### CRSwNP Patients with AR Exhibit Distinct Clinical and Histopathological Features

**Figure 1:**
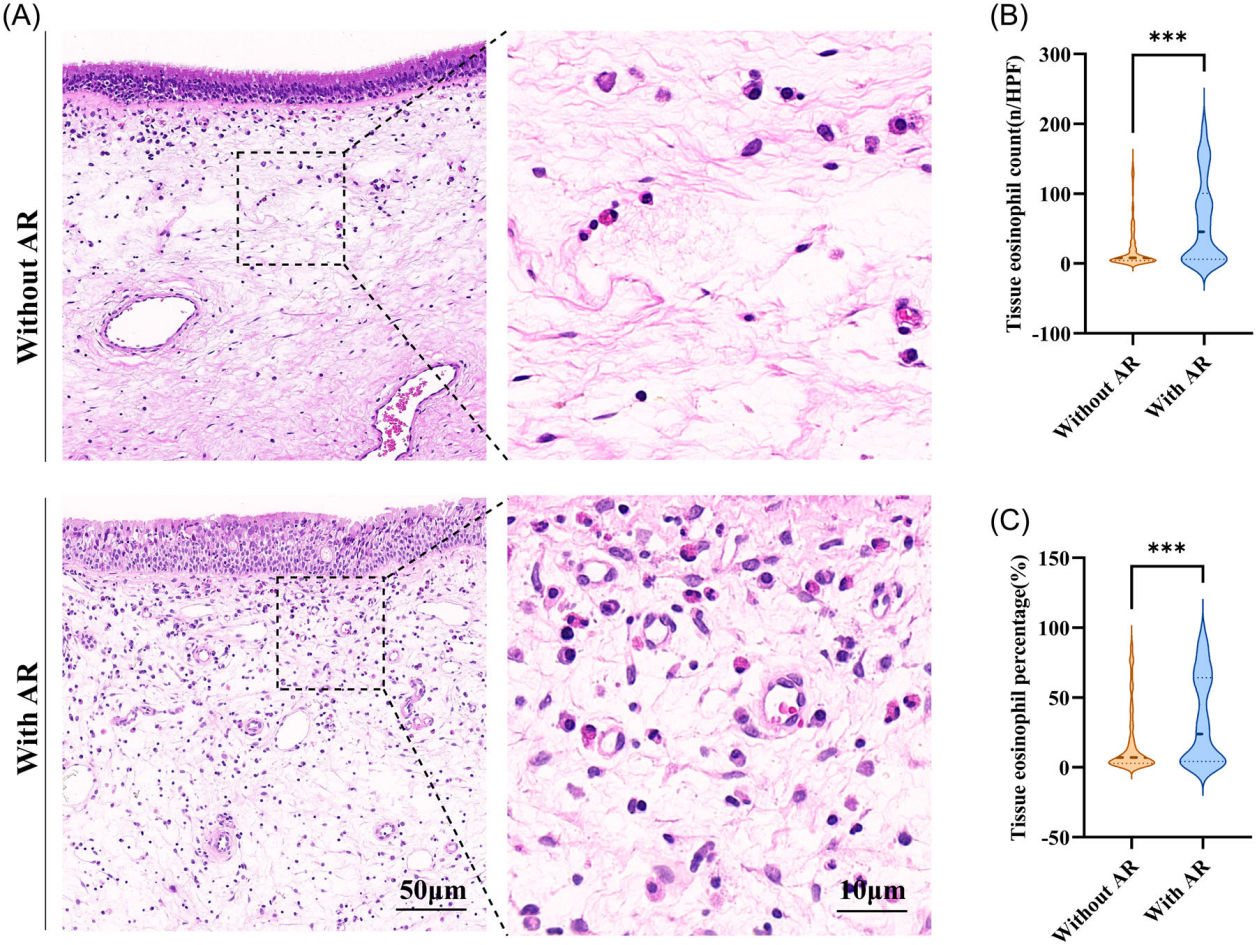
Degree of eosinophilic infiltration in nasal polyp tissues from CRSwNP patients. (A) H&E staining. (B‐C) Comparison of tissue eosinophil counts and percentages between groups. ***P < .001. CRSwNP, chronic rhinosinusitis with nasal polyps; H&E, hematoxylin and eosin.

A total of 603 patients diagnosed with CRSwNP were enrolled in the study, including 202 patients with comorbid AR (with AR group) and 401 patients without AR (without AR group). Compared to the non‐AR group, patients in the AR group exhibited significantly higher rates of previous sinus surgery and asthma (both *P* < .001; Table 1), along with a shorter follow‐up duration and a substantially increased postoperative recurrence rate (*P* < .001; Table 1). Peripheral blood eosinophil count and percentage did not differ between the two groups; however, tissue eosinophilic infiltration was substantially greater in patients with AR (*P* < .001; Table 1). Representative histopathology corroborated more prominent eosinophil accumulation in nasal polyps from AR patients (Figure 1). Collectively, these findings indicate that AR is associated with a more pronounced tissue eosinophilic phenotype in CRSwNP.

**Table 1:** Baseline Clinical Characteristics of CRSwNP Patients With and Without AR

Variables	Total (n = 603)	CRSwNP with AR (n = 202)	CRSwNP without AR (n = 401)	*P* value
Male, n (%)	344 (57.1%)	111 (54.9%)	233 (58.1%)	.460
Age (years)	43.0 (31.0, 53.0)	44.0 (34.0, 54.0)	42.0 (30.0, 53.0)	.105
BMI (kg/m²)	23.9 (21.3, 26.0)	24.1 (21.6, 26.2)	23.6 (21.1, 25.8)	.077
Disease duration (months)	36.0 (24.0, 96.0)	48.0 (24.0, 103.5)	36.0 (15.0, 96.0)	.053
Previous surgery, n (%)	155 (25.7%)	71 (35.2%)	84 (20.9%)	**<.001**
Asthma, n (%)	118 (19.7%)	81 (40.1%)	37 (9.2%)	**<.001**
Peripheral blood eosinophil count (10⁹/L)	0.2 (0.1, 0.3)	0.2 (0.1, 0.4)	0.2 (0.1, 0.3)	.062
Peripheral blood eosinophil percentage (%)	3.3 (1.7, 5.8)	3.7 (1.9, 5.9)	3.1 (1.6, 5.6)	.158
Tissue eosinophil count (n/ HPF)	10.0 (5.0, 56.0)	45.5 (6.0, 100.5)	8.0 (4.0, 29.5)	**<.001**
Tissue eosinophil percentage (%)	8.8 (3.3, 44.8)	23.9 (4.2, 64.1)	7.1 (3.0, 23.5)	**<.001**
Lund‐MacKay score	14.0 (12.0, 16.0)	14.0 (12.0, 16.0)	14.0 (11.0, 16.0)	.086
Lund‐Kennedy score	6.0 (5.0, 7.0)	6.0 (5.0, 8.0)	6.0 (5.0, 7.0)	.339
Follow‐up duration (months)	36.0 (24.0, 48.0)	36.0 (20.0, 36.0)	36.0 (36.0, 48.0)	**<.001**
Postoperative recurrence, n (%)	211 (35.0%)	97 (48.0%)	114 (28.4%)	**<.001**

Bold values indicates statistically significant at *p* < 0.05.

### Comorbid AR as an Independent Risk Factor for Postoperative Recurrence in CRSwNP

To assess the potential role of AR in postoperative recurrence of CRSwNP, we conducted a 3‐year follow‐up of all patients and categorized them into the recurrence group (n = 211) and the non‐recurrence group (n = 392). Comparative analysis revealed that the recurrence group had a significantly higher proportion of patients with comorbid AR, along with markedly increased tissue eosinophilic infiltration (*P* < .001, Table 2). This group also demonstrated significantly elevated peripheral blood eosinophil counts and percentages (*P* < .001, Table 2), a greater incidence of previous sinus surgeries (*P* = .001, Table 2), and higher Lund‐MacKay scores (*P* = .007, Table 2), as well as an earlier onset of recurrence (*P* < .001, Table 2). In contrast, no significant differences were found between the two groups with respect to sex, age, BMI, disease duration, comorbid asthma, or Lund‐Kennedy scores.

**Table 2:** Comparison of Clinical and Histopathological Characteristics Between Recurrent and Nonrecurrent CRSwNP Patients

Variables	Recurrence (n = 211)	Nonrecurrence (n = 392)	*P* value
Male, n (%)	122 (57.8%)	222 (56.6%)	.779
Age (years)	45.0 (33.0, 54.0)	42.0 (30.0, 53.0)	.178
BMI (kg/m²)	24.0 (21.5, 26.0)	23.7 (21.2, 26.0)	.420
Disease duration (months)	36.0 (24.0, 96.0)	36.0 (24.0, 96.0)	.984
Previous surgery, n (%)	72 (34.1%)	83 (21.2%)	**.001**
Asthma, n (%)	49 (23.2%)	69 (17.6%)	.097
AR, n (%)	97 (46.0%)	105 (26.8%)	**<.001**
Peripheral blood eosinophil count (10⁹/L)	0.3 (0.1, 0.4)	0.2 (0.1, 0.3)	**<.001**
Peripheral blood eosinophil percentage (%)	3.9 (2.1, 7.0)	3.0 (1.6, 5.1)	**<.001**
Tissue eosinophil count (n/ HPF)	40.0 (5.0, 100.0)	8.0 (4.0, 28.8)	**<.001**
Tissue eosinophil percentage (%)	25.3 (6.6, 67.1)	6.0 (2.8, 22.8)	**<.001**
Lund‐MacKay score	14.0 (12.0, 16.0)	14.0 (11.0, 15.8)	**.007**
Lund‐Kennedy score	6.0 (5.0, 8.0)	6.0 (5.0, 7.0)	.944
Follow‐up duration (months)	20.0 (14.0, 24.0)	39.0 (36.0, 48.0)	**<.001**

Bold values indicates statistically significant at *p* < 0.05.

Abbreviation: CRSwNP, chronic rhinosinusitis with nasal polyps.

**Figure 2:**
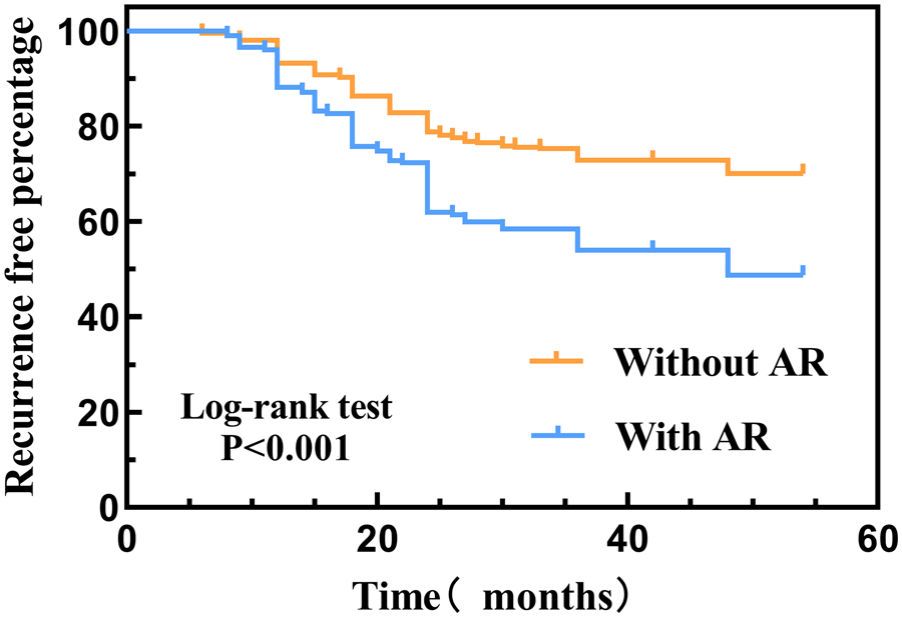
Kaplan‐Meier survival curves showing that AR increases the risk of postoperative recurrence in patients with CRSwNP. CRSwNP, chronic rhinosinusitis with nasal polyps.

Multivariate Cox regression analysis revealed that AR remained an independent predictor of recurrence across all models, including the fully adjusted model accounting for demographic variables, clinical characteristics, eosinophil indices, and radiological scores (*P* = .009, Table 3). Kaplan‐Meier survival analysis further confirmed that patients with AR had earlier onset of recurrence and significantly reduced recurrence‐free survival (*P* < .001, Figure 2). These results collectively support the conclusion that comorbid AR is a robust and independent risk factor for postoperative recurrence in CRSwNP.

**Table 3:** Cox Regression for Postoperative Recurrence in CRSwNP

Variables	HR	95% CI	*P* value
Previous surgery	1.278	0.910‐1.680	.105
AR	1.472	1.103‐1.966	**.009**
Peripheral blood eosinophil count	1.101	0.314‐3.855	.881
Peripheral blood eosinophil percentage	1.068	0.984‐1.160	.117
Tissue eosinophil count	1.003	1.000‐1.006	.090
Tissue eosinophil percentage	1.008	1.002‐1.013	**.009**
Lund‐MacKay score	1.055	1.009‐1.102	**.018**

Bold values indicates statistically significant at *p* < 0.05.

Abbreviations: CI, confidence interval; CRSwNP, chronic rhinosinusitis with nasal polyps; HR, hazard ratio.

### Tissue Eosinophilia Links AR to Increased Recurrence Risk of CRSwNP

**Figure 3:**
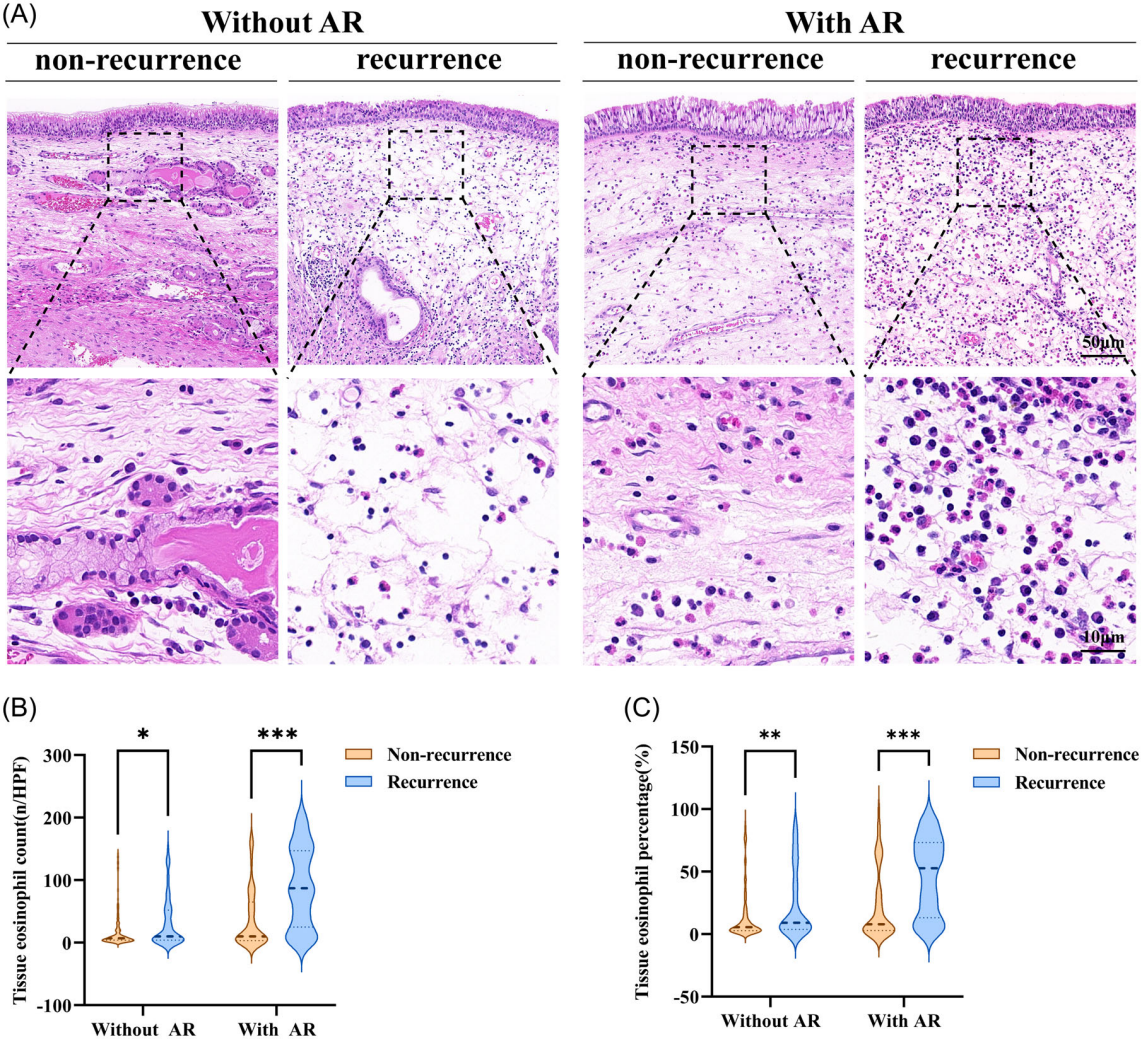
Comparison of tissue eosinophilic inflammation between recurrent and nonrecurrent CRSwNP patients. (A) H&E staining. (B, C) Comparison of tissue eosinophil counts and percentages between groups. *P < .05, **P < .01, ***P < .001. CRSwNP, chronic rhinosinusitis with nasal polyps.

Given the central role of eosinophilic inflammation in CRSwNP pathogenesis, we further explored whether the observed association between AR and postoperative recurrence was mediated by local eosinophilic infiltration. As demonstrated in Table 3, increased tissue eosinophil percentages were independently associated with a higher risk of recurrence. Stratified analysis of the recurrence group revealed that patients with comorbid AR exhibited significantly greater eosinophilic infiltration in nasal polyps than those without AR (Figure 3), with both eosinophil counts and proportions markedly elevated. These findings are consistent with earlier comparisons showing significantly higher tissue eosinophil levels in AR patients overall (Table 1). Notably, even after adjusting for tissue eosinophil indices and radiological severity in multivariate Cox models, AR remained a significant predictor of recurrence, although its effect size was attenuated (*P* = .005, Table 4). Collectively, these results suggest that the pro‐recurrence effect of AR in CRSwNP is largely mediated by enhanced local eosinophilic inflammation, which may serve as a critical pathological mechanism linking AR to disease relapse.

**Table 4:** Cox Regression Analysis of the Impact of AR on Postoperative Recurrence in CRSwNP

Model	HR	95% CI	*P* value
Unadjusted	1.920	1.464‐2.518	**<.001**
Model 1	1.907	1.454‐2.502	**<.001**
Model 2	1.836	1.369‐2.462	**<.001**
Model 3	1.550	1.141‐2.104	**.005**

Bold values indicates statistically significant at *p* < 0.05.

Model 1: Adjusted for age and sex. Model 2: Adjusted for age, sex, BMI, disease duration, prior surgery, and asthma. Model 3: Adjusted for age, sex, BMI, disease duration, prior surgery, asthma, peripheral blood eosinophil count and percentage, tissue eosinophil count and percentage, Lund‐MacKay score, and Lund‐Kennedy score.

## Discussion

In this large retrospective cohort with long‐term follow‐up, we demonstrated that comorbid AR defines a distinct CRSwNP subgroup characterized by enhanced tissue eosinophilic inflammation and a substantially higher risk of postoperative recurrence. The association between AR and recurrence remained robust after adjusting for multiple clinical and pathological variables, underscoring its independent predictive value. Importantly, stratified analyses revealed that among patients who relapsed, those with AR displayed significantly greater tissue eosinophil infiltration than those without AR, whereas no such difference was observed in the nonrecurrence group. These findings collectively suggest that AR may drive recurrence predominantly through amplification of local eosinophilic inflammation, highlighting a key mechanistic link between comorbid AR and adverse surgical outcomes in CRSwNP.

Eosinophilic infiltration is one of the most characteristic type 2 inflammatory phenotypes in CRSwNP, associated with a greater chronic inflammatory burden and a higher propensity for postoperative recurrence.^22^, ^23^, ^24^ Previous studies have shown that activated eosinophils predominate in nasal polyp tissue, releasing various cytotoxic proteins and inflammatory mediators that promote tissue damage and mucosal remodeling, thereby driving disease recurrence.^25^ Our study further confirms that in CRSwNP patients with comorbid AR, the increased risk of recurrence is closely linked to augmented local eosinophilic inflammation. AR may amplify eosinophilic inflammation through multiple mechanisms. First, eosinophilic CRSwNP is often characterized by elevated local tissue IgE levels, particularly against inhalant allergens such as house dust mites, which are more commonly observed in patients with AR. Allergen‐bound IgE can activate mast cells to release histamine, leukotrienes, and other pro‐inflammatory mediators, thereby recruiting eosinophils and amplifying the type 2 inflammatory response.^26^ In addition, AR can further drive Th2/IL‐5‐mediated eosinophilic inflammation, leading to mucosal edema and excessive mucus production. These changes promote sinus outflow obstruction, tissue hypoxia, and microbial dysbiosis, collectively aggravating mucosal inflammation.^27^ Moreover, AR may compromise epithelial barrier integrity, facilitating the penetration of allergens and pathogens and stimulating epithelial cells to release “alarmins” such as IL‐25, IL‐33, and thymic stromal lymphopoietin, which further promote eosinophil infiltration and type 2 immune responses. Notably, eosinophils themselves secrete mediators such as TGF‐β and MMP‐9 that disrupt epithelial integrity, establishing a bidirectional interaction that further amplifies type 2 inflammation.^28^, ^29^

Both animal and human studies have corroborated these mechanisms, demonstrating that AR significantly amplifies sinonasal inflammation, characterized by enhanced eosinophilic infiltration and increased inflammatory markers.^30^, ^31^ Taken together, these findings indicate that AR may establish a vicious cycle involving eosinophilic inflammation, epithelial barrier dysfunction, and local microenvironmental changes, thereby reinforcing an interconnected inflammatory network that accelerates disease progression and recurrence in CRSwNP. It is worth noting that although numerous studies have identified asthma as a predictor of CRSwNP recurrence,^32^, ^33^ our cohort did not show asthma to exert a significant independent effect on postoperative outcomes. This may partly reflect the inclusion of patients with well‐controlled asthma, in whom the potential impact on recurrence was attenuated. Indeed, prior evidence suggests that only in cases of moderate‐to‐severe or poorly controlled asthma does persistent lower airway inflammation exert a “united airway” effect that influences nasal mucosa and elevates recurrence risk.^34^, ^35^ By contrast, AR has a more direct and sustained impact on nasal mucosal sensitization and type 2 inflammation, which may explain why AR emerged as a more consistent pathological driver of recurrence in our study.

Previous studies have indicated that perennial allergen sensitization, such as to house dust mites or molds, shows a stronger association with CRS than seasonal allergy.^36^, ^37^ Moreover, polysensitization is more frequently observed in CRSwNP patients and may reflect a more complex immunological phenotype with enhanced inflammatory activity.^38^ In our cohort, although we did not perform detailed classification of allergen sensitization patterns, most participants were from southern China, where dust mites and molds are the predominant allergens, and specific IgE testing confirmed that sensitization was mainly directed against these perennial allergens.^39^, ^40^ Therefore, the majority of AR patients in this study likely exhibited a perennial AR phenotype, which may partly account for the stronger link between AR and CRSwNP recurrence risk observed in our findings.

This study has several limitations. First, as a single‐center retrospective cohort study, selection bias cannot be completely excluded, and the retrospective design inherently limits causal inference regarding the association between allergic rhinitis and postoperative recurrence of CRSwNP. Second, the determination of recurrence relied on endoscopic findings and the assessment of patient‐reported symptoms, which may be subject to inter‐observer variability and could introduce a certain degree of classification bias. In addition, the absence of systematically collected standardized nasal polyp scores, radiologic scores, and patient‐reported outcome measures may have limited a more granular assessment of disease severity. Furthermore, subgroup analyses based on allergen sensitization patterns were not performed, restricting assessment of their potentially differential effects on recurrence risk. Finally, this study primarily focused on clinical and histopathological associations and did not explore in depth the underlying pathological mechanisms through which allergic rhinitis influences tissue characteristics in CRSwNP, which warrants further investigation in future prospective and mechanistic studies.

## Conclusion

This study demonstrates that CRSwNP patients with comorbid AR represent a distinct subgroup characterized by enhanced tissue eosinophilic inflammation and an elevated risk of postoperative recurrence. Further analyses indicate that AR may promote recurrence primarily through amplification of local, rather than peripheral, eosinophilic inflammation. These findings underscore the importance of intensified postoperative surveillance and tailored management strategies for patients with comorbid AR to mitigate recurrence risk.

## Author Contributions

**Wei Zhong**, data collection, statistical analysis, drafting of the manuscript; **Shaobing Xie** and **Haijun He**, final statistical review, acquisition, analysis, or interpretation of data; **Hua Zhang**, study concept and design, statistical analysis; **Weihong Jiang**, administrative support, final statistical review; **Can Liao**, acquisition, analysis, or interpretation of data; **Zhihai Xie**, study concept and design, final review of manuscript.

## Disclosures

### Competing interests

None.

### Funding source

This research was supported by the National Natural Science Foundation of China (No. 82371126, No. 82371127), the Natural Science Foundation of Hunan Province (No. 2023JJ30953), the Open Funding of State Environmental Protection Key Laboratory of Monitoring for Heavy Metal Pollutants (No. KLMHM202404).
